# Generativity in Creative Storytelling: Evidence From a Dementia Care Community

**DOI:** 10.1093/geroni/igaa002

**Published:** 2020-03-28

**Authors:** Seoyoun Kim, Kyong Hee Chee, Olga Gerhart

**Affiliations:** 1 Department of Sociology, Texas State University, San Marcos; 2 Department of Philosophy, Texas State University, San Marcos

**Keywords:** Generativity model, Narrative identity, TimeSlips

## Abstract

**Background and Objectives:**

Creative group storytelling as utilized in TimeSlips is a social activity that focuses on communication, improvisation, and creativity among its participants with dementia. A collective narrative is a channel through which participants express themselves, and it thus signifies clues about their identities, values, and experiences. No study to date, however, has examined the contents of the stories. Using the generativity model as a theoretical underpinning for analysis, this paper examines the emergent themes of such collective stories.

**Research Design and Methods:**

This study used the data collected in a memory care community where a research team recruited and engaged 21 out of approximately 80 residents with dementia in 6 weekly creative storytelling sessions; 4 small groups of 4 to 6 participants created a total of 24 collective stories. Three researchers analyzed these stories by open-coding emergent themes. Once coded manually, the narratives were managed and analyzed in NVivo.

**Results:**

Consistent with the concept of generativity, a thematic analysis of collective narratives revealed various aspects of participants’ generative concerns. Three themes related to generativity: (1) caring and promoting the well-being of others, (2) family values, and (3) positivity.

**Discussion and Implications:**

The narratives show that participants living with dementia continue to express their generative values and concerns. The findings reveal generative identities held by persons living with dementia, which help destigmatize dementia. The findings also shed light on why creative group storytelling may affect multiple positive outcomes for its participants.

**Translational Significance:** This 1-year project in partnership with a memory care facility sought to find generative themes in the narratives from a creative group storytelling program. Three overarching themes were identified across 24 collective stories. The findings clearly show generative desires and concerns of the participants of the program and highlight the significance of continued engagement activities for persons living with dementia that fulfill their fundamental human needs such as generativity.

## Background and Objectives

Dementia affects an estimated 5 million people in the United States, and this number is expected to rise to 14 million by 2050 (Centers for Disease Control and Prevention, 2018). One of the programs for persons living with dementia is an arts-based, creative storytelling program (CSP) called TimeSlips. During CSP, a facilitator encourages participants to exercise their imaginations by creating a freeform story from staged pictures (Basting, 2011). Unlike most individual-targeted programs, CSP provides a unique avenue for the participants to collectively create an end-product (i.e., story) and engage in meaningful social interactions. Indeed, storytelling as a social activity focuses on communication, improvisation, and collective creativity (Basting, 2011), and has shown to improve relationship quality (Vigliotti, Chinchilli, & George, 2018), communication (Fritsch et al., 2009; Phillips, Reid-Arndt, & Pak, 2010), alertness (Fritsch et al., 2009), and mood (Phillips et al., 2010). The activity is also known for improving well-being by creating a social role (Whitehouse & George, 2008).

The research findings concerning CSP suggest the significance of meaningful social roles, creative contribution, and collective goal pursuit for participant experiences (Kontos, Miller, & Kontos, 2017). Though many empirical studies have investigated the salutary effects of CSP (e.g., Fritsch et al., 2009; George & Houser, 2014), scant attention has been paid to the contents of actual stories. These stories contain expressions of or clues about storytellers’ identities, values, and emotions. In fact, Basting (2003) noted that one can learn about the storytellers’ lives through the stories. Collective narratives (i.e., stories) may reveal psychological, social, and cultural aspects of storytellers’ lives beyond mere stories of fictional characters (Nyman & Szymczynska, 2016).

Multiple theoretical perspectives explain the benefits of creative storytelling, such as a theory of cognition (Koestler, 1964), a relationship-centered care model (Adams & Gardiner, 2005), and a narrative citizenship model (Baldwin, 2008). We use the generativity model to examine collective stories. Generativity represents a desire and active involvement of self to promote the well-being of others and oneself, expressed through parenting, mentoring, and maintaining meaningful relationships. Canonical generativity scholars emphasize lifelong efforts of generativity (Erikson, Erikson, & Kivnick, 1986; Kivnick & Wells, 2014), but the dominant framework tends to be biased toward the cognitively able. Research shows that those affected by dementia maintain desires and concerns to meaningfully contribute to the community even in the absence of family members and younger generations (Doyle, Rubinstein, & de Medeiros, 2015; Fritsch et al., 2009). Various social engagement programs and other generative opportunities may fulfill these desires, which calls for an appreciation of participants’ capacity to create, think, and express themselves (Dupuis, Kontos, Mitchell, Jonas-Simpson, & Gray, 2016). In this regard, CSP enables individuals living with dementia to collectively create something that is of value to self and others within a supportive environment. The current paper seeks emergent generative themes from the stories in order to examine generativity as an underlying mechanism linking collective storytelling with purported health benefits, quality of life, communication, and cultural enrichment.

## Generativity in Persons Living with Dementia

Initially developed by Erik Erikson (1950) as a midlife task, the concept of generativity has evolved over time. Kotre’s (1984) alternate definition considers individual needs regardless of age, life stages, or roles. Erikson and colleagues later echoed that the grand-generative functions in later life might help people to stay truly alive (Erikson et al., 1986). Scholars recognize various dimensions and expressions of generativity throughout the life course (Clark & Arnold, 2008; Keyes & Ryff, 1998), effectively rendering generativity as a construct. Kim, Chee, and Gerhart (2017) recently redefined generativity as “the human experience of contributing to and promoting the lives of others and oneself” (p.2). This definition fits the empirical findings that show similar levels of generativity across the life course (McAdams & de St. Aubin, 1992), regardless of marital or parental status (Rothrauff & Cooney, 2008), for prior generations (e.g., filial caregiving; Peterson, 2002), and for self-fulfillment (Rubinstein, Girling, de Medeiros, Brazda, & Hannum, 2015).

Concerning antecedents and contexts of generativity, McAdams and de St. Aubin’s (1992) model comprises psychosocial features linked with generative outcomes: cultural demands (e.g., cultural expectations) and inner desires serve as external and internal motivational sources, promoting generative concerns for others. These facilitate generative behaviors expressed by creating, maintaining, and offering tangible and intangible products/value for others and oneself. Narratives can reflect generative concerns, commitments, and behaviors situated within the social world. Though not explicated in the original model, the connection between generativity and well-being is supported by a large body of empirical literature across the life course (An & Cooney, 2006; Ardelt, Landes, & Vaillant, 2010; Carragher, 2017; Cheng, 2009; Sabir, 2015; Tabuchi, Nakagawa, Miura, & Gondo, 2015).

Persons living with dementia in later life do not prominently figure in the generativity literature. Cultural demands for generativity are virtually nonexistent for those with low cognitive functions due to the assumption that concerns for others and helping behaviors are predicated on cognitive abilities. Yet growing evidence reveals that one need not rely on critical reasoning to be generative as it has affective and crystallized components such as love, support, and caring (Doyle et al., 2015; Frensch, Pratt, & Norris, 2007; Nyman & Szymczynska, 2016). Persons living with dementia continue to engage in generative behaviors such as helping their friends, maintaining grandparent roles, and caring for others (Doyle et al., 2015). Doyle and colleagues (2015) found that some study participants with moderate to severe dementia served as resident council members and helped relay the voices of other residents. Because memory care facilities tend to focus on disease management, residents’ generative desires and abilities may be overlooked.

## Generativity in the Context of Creative Storytelling

The current paper explores the themes of generativity in the narratives from CSP. CSP enables people with dementia to express themselves without relying on memories by creating a “failure-free” environment (Basting, 2003; Harries et al., 2013). These narratives are shown to be both self-reflective and creative (Kontos et al., 2017). They may reflect generative concerns, commitment, and behaviors situated within the social world (McAdams & de St. Aubin, 1992). Collective narrative is a channel through which participants express themselves and thus signifies clues about their identities, values, and experiences. In that regard, CSP is a viable avenue for creating and maintaining a tangible product (i.e., a story) that facilitates participants’ senses of generativity and belonging (Kontos et al., 2017).

Persons living with dementia are marginalized from both the conceptualization and measurement of generativity due to the dominant medical and care models that consider them care recipients rather than active participants of society. In particular, self-reported measures of generativity assume that respondents’ short-term memories are intact and hence do not effectively assess generative concerns and behaviors. Thus, it is important to consider alternative methods to explore generativity in this population and reflect diverse expressions of generativity.

Offering a few lines to a story during a CSP session can be a generative experience for participants regardless of cognitive ability. Thus, CSP provides a suitable avenue for exploring generativity among persons living with dementia. Finding generative themes could help highlight participants’ creative and generative potentials rather than cognitive declines. In the context of CSP, then, it may be possible to alter the typical stereotypes of a person with dementia as one who only receives help from others.

## Purpose

Based on the empirical evidence of storytelling benefits and the generativity model, the current paper explores themes of generativity in the narratives created during collective storytelling sessions. The study will extend previous empirical research on TimeSlips group storytelling by identifying generative expressions among persons living with dementia in their collective stories. There has been no study to date that has examined the contents of the collective narratives.

## Method

Researchers analyzed the narratives collected for a larger funded project in partnership with a memory care community located in Texas. The Institutional Review Board at Texas State University approved the study.

## Participants and Recruitment for the Project

Before recruiting participants, the authors and the graduate research assistant visited the site and gave a demonstration storytelling session. The researchers communicated with potential study participants and their family members via face-to-face contact and electronic mail. Of approximately 80 residents at the site, the researchers were able to recruit and obtain consents from 21 residents and their family members (15 in Fall 2018 and 6 in Spring 2019). Excluding residents with very limited functional ability (i.e., needing total help with getting in and out of bed, bathing, and eating), four groups were formed (Group A = 6 participants, B = 5, C = 4, D = 6). During the first storytelling session for each group, basic participant demographic information (e.g., sex, health) was collected by the formal care partners.

## Procedures

The storytelling sessions were conducted between September 2018 and April 2019. Three groups participated in the Fall of 2018, and one group in the Spring of 2019. The sessions were offered once a week for 6 weeks and took place in a private group meeting room, so only scheduled participants could attend. Participants’ wishes to attend each session were honored. During the storytelling program, a TimeSlips-trained facilitator showed a picture and asked open-ended questions in reference to the picture. A total of 13 pictures were selected and used in consultation with formal and informal care partners to avoid triggering negative reactions. Examples of questions included: What do you see/smell/hear in the picture? What is happening in the picture? Is there anything (anyone) we don’t see in the picture? What are they thinking (saying)? What is going to happen next? All verbalization was videotaped and incorporated by a scribe into a free-form story for further analyses. The scribe did not edit any part of the stories: grammatically incorrect sentences, contradicting storylines, and nonexistent words were incorporated into a story. At the end of the program in the spring of 2019, all 24 stories were compiled into a booklet and returned to participants for a record of their communal effort.

Each of the four groups created six stories, resulting in 24 stories in total. Each session lasted from 30 min to an hour (mean = 41.21 min). Each story consisted of 1–3 double-spaced pages of content. Participants’ demographic characteristics are presented in Table 1. We do not know the ages of the participants, but approximately 85% of the residents at the site were over the age of 65. Attendance for the storytelling sessions averaged 68% overall (Group A: 50%–100%, B: 80%–100%, C: 66%–100%, D: 57%–100%).

**Table 1.** Study Participants and Storytelling Session Characteristics

	Mean/Percentage	Range
Study participants (*N* = 21)		
Female (%)	70%	—
Length of stay	2.06	1–3
Self-rated health*	3.52	1–5
MMSE scores	14.58	0–28
Storytelling sessions (*N* = 24)		
Length (in minutes)	41.21	28.39–58.29
Attendance (%)	68%	40%–100%

*Note*: MMSE = Mini-Mental State Examination.*As reported by formal care partners.

## Analysis

Although the generativity model framed the study, the first and second authors and the graduate assistant initially open-coded all stories in order to capture all themes in order to note any emerging categories (Erlandson, Harris, Skipper, & Allen, 1993; Luborsky, 1994). The coding team met weekly for debriefing and building consensus (Erlandson et al., 1993). Frequent and significant open codes helped identify the most relevant focused codes in NVivo (version 10). The codebook included 12 codes which were subsequently collapsed into three themes, each exemplifying the concept of generativity. We counted the number of stories that exhibited each of the themes. Through the entire iterative process, the coding team kept extensive notes and multiple codebook files related to analytic decisions and consulted the third author in order to reach consensus (Guest & MacQueen, 2008).

## Results

A thematic analysis of stories revealed various aspects of participants’ identities, values, past experiences, and autobiographical memories. Specifically, multiple generativity themes were identified: (1) caring and promoting the well-being of others, (2) family values, and (3) positivity. People (spouse, children, other family members, friends), animals (dogs, cats, birds), and objects (picture, books) emerged as vectors for generativity. Throughout the remainder of this paper, the words “participants” and “storytellers” are used interchangeably.

### Theme 1: Caring and Promoting the Well-being of Others

At the core of the generativity concept are caring and concern for others (Frensch et al., 2007) and oneself (Kim et al., 2017), and the collective stories reveal that the most prominent theme is how people care about others. Nineteen collective stories showed various aspects of this theme. Participants particularly expressed concerns for the safety, well-being, relationship quality, and happiness of the characters in the pictures. Children, spouses, siblings, friends, and animals were identified as vectors of caring. The most common descriptors included protecting, taking care of, watching over, being careful, thinking about, helping, and loving. When participants saw a picture of a dog and a little girl sleeping in bed, they asserted, “[The dog] is taking care of the little girl. The dog is looking over her … making sure she’s happy and safe. Animals are quite intuitive. They have the ability to make people feel better … The little girl’s family is trusting of the dog and perhaps has trained the dog to guard the little girl” (Group C, Story 5). When asked what happens next, the participants pointed out that “the mother makes the dog get out of the room, go to another room or go outside. The child needs to sleep more.”

When the picture of two women facing each other was shown, the participants identified them as mother and daughter. They elaborated that “the mother is protecting her … The daughter is sitting in the chair and they are holding onto each other” (Group A, Story 3). Another group offered, “one lady is giving information to the younger one just to keep her in touch … [and] Mom’s trying to soothe her and make her feel better” (Group C, Story 1). Notably, these storytellers initially described the two women as friends and then later as mother and daughter.

In Story 4 by Group D, the picture portrayed an older woman and a child with a computer. The participants quickly described that “her grandparents are teaching her the alphabet. They are helping her.” When prompted what happens next, they created a story that “the lady is going to take her to get some milkshake or whatever the girl wants.” In general, participants frequently indicated that the characters in the picture need to be cared for (e.g., fed, dressed, kept out of trouble, protected, and loved) by somebody, especially a parental figure.

### Theme 2: Family Values

As Kotre (1984) demonstrated, parenting or raising family is a prominent generative concern for older adults. Parental generativity is expressed through nurturing and caring for children. Family values were discussed in 20 collective narratives. When identifying the relationship among characters, mother, father, sister, brother, child(ren), family, and baby (infant) were the most frequently used words to describe any character, even if the pictures did not clearly depict family relationships. Further, in nine stories, storytellers created a family member character not shown in the picture.

Based on a picture of a woman and three small children, participants noted, “Children can be challenging because you can’t just sit around and let them grow … the mother’s having a job. When there are multiple kids at home, then you have to figure out an activity that suits her.” They added, “There has to be a father somewhere. The father usually has his child on the lap. They [the family] look calm and nice and that’s a factor for a successful family” (Group A, Story 5).

The storytellers once actively discussed what mothers do while looking at a picture of five persons holding hands in a circle. They initially created a story about a family of mother, father, an older child, and a younger child, but the discussion quickly evolved to explain the person who took the photo. At the end of the story, they stated, “There are two girls and the daddy … The picture is taken by the mom, because that is what she does. The holding of the memories [is why they took the picture]” (Group D, Story 3).

Together, the storytellers revealed their familial values either by contributing to the storyline or creating a family character that is “missing” from the given picture, such as the father, mother, grandparent, and siblings.

### Theme 3: Positivity

Generative motivations imply a desire to create and maintain any artifacts deemed “good” (Peterson & Stewart, 1996). Particularly among older adults, communal motives such as making a lasting legacy increase in predicting generativity (Rubinstein et al., 2015). Throughout the program, the storytellers were concerned with creating a “positive” story. Twenty-one stories revealed this theme. Some of the most frequently used positive descriptors included: cute, beautiful, nice, good job, good time, happy, wonderful, well (e.g., doing well, going well), and joy. This theme usually appeared towards the end of the story.

In one story, participants created a narrative in which the characters are “all together and being nice together.” When prompted what was going to happen next, they mentioned that “they are going to have a good day all Saturday because it isn’t raining” (Group A, Story 6). In another story, participants asserted that they “see togetherness between the two people.” After naming the two characters Laura and Mary, they concluded that “they live happily ever after” (Group B, Story 5). One especially encapsulates this theme. The picture shown was of three men sitting around the table and in the middle of a conversation.The picture projects excitement, joy, and life. Life is creamed, not curdled. They are old, past the age of joy, but they look happy … They are looking forward to the future … They are still excited about their life, what’s next and what they can do to improve in their own lives. It’s the human spirit, the anticipation in life. Just because the skin has gotten frail, it doesn’t mean our brain, spirit has gotten frail. The privilege is the participation in life. (Group C, Story 6)

Another story illustrates an interesting positivity theme: When shown a picture with two female characters, participants stated, “They seem to be doing well … it should take place somewhere pleasant. I think it should express joy and happiness. It’s peaceful” (Group C, Story 4). During this story session, storytellers were interested in creating a positive story for a potential audience, even though they were told that their identities would remain anonymous.

There were also some less prevalent generativity-related themes. Themes related to teaching values and knowledge (Rothrauff & Cooney, 2008) were apparent in five stories. Some stories directly reflected values and knowledge of the participants and their effort to impart their wisdom. Other stories included a description of teaching or guiding behaviors, such as teaching alphabets to children, teaching someone how to cook, and giving information to someone in the picture. A desire to maintain and cherish tradition, another key feature of generativity (Vaillant, 1995), was shown in four stories. For example, during one session, participants were given a picture with two female characters gazing at each other. One character was holding an inanimate object. Participants collectively decided that they were mother and daughter conversing about a picture. They shared, “They are cherishing the picture … and discussing what is similar and what is different … what is similar is the fact that mothers and daughters still love each other. It’s loving, and the daughter is feeling loved” (Group C, Story 4).

## Discussion and Implications

Guided by the generativity theory, the current paper examined generative themes in the collective stories of residents at a memory care facility. This study identified three overarching themes, including caring for others, family values, and positivity. Despite the popular belief that people living with dementia are considered merely the recipients of care, the findings show that participants continue to express concern for others, continued family values, and create and maintain positive objects (i.e., stories). The findings echo previous case studies that showed older adults’ continued engagements in generative acts during cognitive decline (Doyle et al., 2015). It also extends the literature by showing that generativity is likely a commonly held value among persons living with dementia, as reflected in 24 collective stories.

The current findings have implications at multiple levels. First, the findings highlight diverse expressions of generativity in persons living with dementia. Participants expressed their generative concerns and desires toward and through the characters in the story. In line with the generativity model (McAdams & de St. Aubin, 1992) and Erikson’s concept of generativity versus stagnation (1950), the act of creating a collective story may serve as an opportunity to exercise generativity for persons living with dementia. Concerns for others evidenced in CSP may indicate participants’ continued development as humans.

Second, the findings may shed theoretical light on the benefits of CSP. The narrative citizenship literature emphasizes that the ability to express a person’s identity and citizenship *as well as* the structural opportunity to exercise one’s citizenship are critical (Baldwin, 2008; Dupuis et al., 2016). CSP may be beneficial because participants can voice their stories and coconstruct a narrative. Further, sharing the stories with other residents and staff members at the facility may reinforce care partners’ understanding of their residents, which, in turn, may improve quality of care and a sense of belonging (Kontos et al., 2017).

Third, more opportunities for generative acts could be offered to persons living with dementia. A few engagement staff members at the research site noted that they were impressed by how their residents performed during CSP and that their observation of the storytelling sessions helped them better understand the residents, including what their experiences might have been like, how much they enjoyed storytelling, and how important it was for them to be respected. This implies that they began to regard the residents in terms of their abilities rather than deficits. It is relatively easy to create small opportunities for the residents to express and realize their generativity. Even though some engagement programs are offered in memory care communities, there is much room for improvement in order to fulfill the members’ fundamental psychological, social, and cultural needs for generativity.

Finally, we offer alternative modes of investigating generativity and related benefits in this population. Future studies can use CSP as a method of collecting meaningful information about persons living with dementia that otherwise may be challenging to obtain because they need to be heard (rather than recalled) for inclusion in program and policy development. Our unpublished findings and forthcoming papers use video-recorded data to show other significant themes in the context of CSP.

The results should be interpreted given the following limitations. First, the current study used data from a small convenience sample and a relatively short-term program. Future studies should include an experimental design in order to compare the contents of the stories and related outcomes among program participants. Second, even though the facilitator attempted to ensure a contribution from every storyteller, it is possible that more vocal participants dominated the process. Third, even though the pictures were selected in consultation with the staff, each picture may have influenced the participants differently.

Despite the limitations, the study contributes to the literature on generativity among persons living with dementia and highlights the contents of the narratives created by the participants based on the theory of generativity. The findings can help the community to acknowledge older adults living with dementia in terms of their generative potentials and, importantly, to destigmatize dementia.
